# The relationship of personal budgets with independence, participation and quality of life; a secondary analysis of survey data using propensity score matching

**DOI:** 10.1186/s41687-020-00258-x

**Published:** 2020-11-07

**Authors:** Anna Maria Marangos, Jurjen Iedema, Mirjam de Klerk, Isolde Woittiez, Peter P. Groenewegen

**Affiliations:** 1 The Netherlands Institute for Social Research, SCP, P.O. Box 16164, 2515 XP The Hague, The Netherlands; 2 Nivel – Netherlands Institute for Health Services Research, P.O. Box 1568, 3500 BN Utrecht, The Netherlands; 3 Utrecht University, Faculty of Geoscience, Faculty of Social Science, P.O. Box 80.115, 3508 TC Utrecht, The Netherlands

**Keywords:** Personal budgets, Social care, Health care, Quality of life

## Abstract

**Objectives:**

Personal budgets for social and health care have been introduced in many European countries over recent decades. The assumption is that people with a personal budget are able to purchase care that matches their needs more closely and therefore experience greater independence and improved well-being. The question is whether this assumption is true. Little research has been carried out on this and the research that has been carried out is inconclusive and hampered by methodological limitations.

**Methods:**

We performed a secondary analysis of data collected in a survey among persons who had submitted an application for social support. Propensity score matching was used to investigate whether people with a personal budget experience better independence, participation in society and quality of life than comparable people using conventionally organised help.

**Results:**

After matching, no significant effects of the personal budget were initially found. A sensitivity analysis that excluded the variable sense of mastery from the calculation of the propensity scores, showed a significantly greater independence for those using a personal budget.

**Conclusion:**

There may be several reasons for this lack of effect. First, perhaps there are no effects. It is also possible that effects can only be found in specific situations and/or specific groups.

## Introduction

Several European countries have introduced a personal budget (PB) for social care and health care in recent decades, including the Netherlands, Germany, France and the United Kingdom. In the literature, large differences between countries have been documented in scope (health care, social support), entitlement (needs and/or means tested), benefits (open allowance or a specific care package), funding (social insurance, taxes) and management (restrictions of use) of these schemes [1–3]. However, what is largely lacking is an analysis of the effects of having a PB on client reported outcomes, such as independence and quality of life. This study analyses data for the Netherlands to assess effects of having a PB on client reported outcomes.

In the Netherlands, a PB for social care and health care is anchored in several laws and is thus an existing right. People with a chronic disease or limitation in functioning who are eligible for support can opt for in-kind delivery (via an institution) or via a PB. With a PB they can hire care providers themselves for the support or care they need. Social support (for example home help) or a budget for this is arranged through municipalities. In the area of health care, PBs exist for personal care and nursing. Until 2015 personal care and nursing care were part of the Exceptional Medical Expenses Act [4]. The data we will use for secondary analysis in this article, were collected in 2012. With a reform of long-term care in 2015 the situation changed. Personal care and nursing care at home is part of the basic health insurance package since 2015. If this personal care or nursing care is substituting for admission to a nursing home it is regulated via the Long-term Care Act, introduced in 2015.

Since people with a PB purchase the help they need themselves, they have an influence over which help is provided, by whom and when. The arguments of policy-makers and advocates in favour of PBs, in social care as well as in health care, are greater freedom of choice, control and tailor-made care [5–7]. A PB is expected to give people a say over who helps them, when and where. They do not have to admit different care providers for different types of support, which many people experience as unpleasant. This, it is argued, leads to greater independence and improved well-being [5, 8]. Sometimes a PB might save costs due to the lower price of care purchased with a PB compared with publicly organised care [9, 10]. This connects to the literature on the commodification of care through cash benefits and its effects on professional care-givers [11–13].

PBs are assumed to have positive effects on independence and well-being, but little research has been carried out to test this assumption. In the Netherlands one qualitative study has been carried out, which concluded that people with a PB experience that having a PB contributes to their quality of life [14], but no quantitative research has been done. Internationally we found only few quantitative studies testing the assumptions. Two important studies concern the evaluations of the pilot programme for individual (social) budgets and the health budgets pilot in England. Part of the national evaluation of the individual budgets pilot programme was a randomised controlled trial [15]. The trial found positive outcomes in the area of perceived control over one’s life in the individual budget group compared to the comparison group. Moreover, effects seem to depend on the user groups; for some user groups outcomes seemed to be positive while for the largest group, older people, no positive outcomes were found [15, 16]. In line with this, Woolham et al. [5] did not find a difference in outcomes for people aged 75 years and over, an important target group of direct payment PBs in England. The analyses in the evaluation of the health budgets pilot revealed a significant positive relationship with care-related quality of life and with mental well-being [17]. No relationship was found with health-related quality of life [18]. A review of – mainly UK – studies found a positive association with quality of life for people with mental health problems, but due to methodological limitations the results are not regarded as sufficiently reliable [19].

In this article we investigate whether PBs in the Netherlands do indeed have positive effects. In contrast to the evaluations of the social care and health care budgets pilot programmes in England [15, 17], the data for our study were collected in an existing situation of PBs. A trial was therefore not possible; however, we address methodological limitations. Our hypothesis is in line with the assumptions underlying the introduction of PBs and with the tentative findings of earlier research, namely that people in receipt of a PB experience more independence, social participation and a higher quality of life than people who receive help in kind. These outcomes reflect the objectives of the principal laws governing social support and care in the Netherlands. Our research question is:

*Are people with a PB more independent, do they participate more in society and do they experience a higher quality of life than comparable people receiving help in kind?*

The ideal research design for answering this question is an experimental approach (randomised controlled trial, RCT), whereby the researcher decides at random who receives a PB and who receives help in kind. In an RCT no other factors influence the relationship between the form of support received and the outcome. However, because of the legislative anchoring of PBs, an experimental approach is not possible in the Netherlands. If recipients of help were nonetheless asked to take part in an RCT, those who agreed to do so would be a selective group. Observational quantitative research is therefore an option for answering our research question. We used data from a survey among people who applied for help with the household, social support, personal care and nursing care, either in kind or in the form of a PB. With propensity score matching, we approximate the experimental design as closely as possible [20].

Dutch research has shown that mainly people with a severe physical disability, a progressive disability, a long-term mental health problem or parents of a child with an intellectual disability are PB-holders [21, 22]. Relatively young people and people with a higher education more often have a PB. Self-selection results in people who are relatively autonomous and self-reliant to prefer a PB [10, 23]. It is therefore not clear whether people acquire more autonomy and become more independent as a result of the PB, or whether PBs are chosen by people with a high degree of autonomy and independence.

A study of applicants for support showed which people with a disability are more or less independent, participate to a greater or lesser extent and have a better or poorer quality of life [22]. People with a severe physical disability, intellectual disability or psychosocial problems more often experience relatively lower independence; people with a severe physical disability, psychosocial problems, higher education or low income perceive their scope for participation more negatively; relatively young people, people with a low income, people with a severe physical disability or long-term mental health problem experience a lower quality of life.

Consequently, some of the same factors which explain the choice of a PB also determine the perceived independence, participation and quality of life. However, the effect does not always operate in the same direction. For example, relatively young people and people with a severe physical disability more often choose a PB, but experience lower quality of life than older people and people with a less severe disability.

## Method

### Data collection and operationalisation

In the period April–June 2012, as part of a national evaluation of the Dutch legislation, face-to-face interviews were held with persons who had submitted an application to their local authority at the end of 2011 for social support. The respondents lived in 73 Dutch municipalities. A total of 5610 people were approached, of whom 4041 participated (response rate 72%). For the secondary analysis reported in this article, we selected all independent community-dwelling adults who had applied for help with the household, social support, personal care and nursing care and who took part in the interview themselves (no proxies). Only this group were asked questions about subjects such as quality of life. The number of respondents for the analyses was 2321. The majority of respondents (*n* = 2025) had a physical disability; 399 respondents (also) had a long-term mental health problem and 77 (also) had an intellectual disability. A total of 2079 respondents (89.5%) were receiving help in kind and 242 (10.4%) had a PB.

The interviews were held in the respondents’ homes using structured questionnaires. During the interviews, respondents were asked what type of support they had applied for and about the support they were already receiving before their application. Applicants were asked whether the support – both that already being received and that for which they had applied – was provided in kind or in the form of a PB. More than two thirds of the respondents who applied for a PB, already had a PB for another form of support or for the same kind of support but needed a re-assessment.

### Measurements

In this article we relate the form of support (PB or help in kind) to the outcome measures independence, participation and quality of life. With regard to independence, respondents were asked to indicate on a scale from 0 to 10 how well they were able to manage with support. For social participation, respondents were asked to indicate on a scale from 0 to 10 to what extent they were able to perform activities such as visiting a club/association or attending a cultural activity. Quality of life was measured by asking respondents how satisfied they were with the lives they were leading. The use of such a 0–10 scale is very common in international research [24] and is regarded as sufficiently reliable and valid [25–27].

We took into account the personal characteristics which have been found to be related to the choice of a PB and/or independence, participation or quality of life, namely household composition, age, sex, education, income, degree of control, severity of physical disability and having a long-term mental health problem. Household composition was categorised into living alone versus cohabiting. Age ranged from 18 to 97 years. Education was divided into eight categories from low (primary education) to high (university). For income, respondents were asked about their net monthly household income and categorised into < 1000, 1000–1500, 1500–2000 and > 2000 euros. A validated mastery scale was used to ascertain respondents’ degree of control [28]. Mastery is the degree to which people feel they have a grip on events and situations, in contrast to feeling that their lives are ruled by chance or by other people [29]. An example of an item on this scale is: “I have little control over things that happen to me”.

To establish the severity of their physical disability, applicants were asked about nine activities of daily living (such as washing or dressing), the household (preparing meals, heavy household work) or mobility (climbing stairs, standing for ten minutes), and requested to indicate whether they could perform these activities without difficulty, with some difficulty or not at all [30–32]. The scale runs from 15 (not or slightly physically disabled) to 45 (severely physically disabled). Whether people suffer from a long-term mental health problem was asked dichotomously (yes/no). Depression was given as an example of such a problem.

The local authority where people can apply for PBs are municipalities. Characteristics of municipalities may influence the application process and its results. We therefore added municipal co-variates: percentage of 65+ living alone, percentage disabled persons and percentage people below the Dutch poverty threshold.

### Analyses

We applied propensity score matching for multilevel analysis [33, 34]. This is a widely used matching method [35] that attempts to estimate the effect of a treatment or intervention by accounting for the covariates that predict receiving the treatment and thus reduces the bias due to potential confounding variables. The treatment here is receiving a PB (‘experimental group’) and receiving care in kind (‘control group’). The propensity score matching is based on the background variables referred to above which were found in earlier research to be associated with the choice of a PB and/or the outcomes. The matching is not absolute but is approximated using a probability, the propensity score. Respondents with the same propensity score are – on average – comparable as regards the measured background variables. We followed the guidelines proposed by Caliendo and Kopeinig [34]. Because respondents were clustered within municipalities, we performed a multilevel analysis [35] with two cross-classified levels: municipalities and the matched pairs resulting from the propensity score matching. First, we checked whether the overlap in the propensity scores of the two groups was sufficient. This proved to be the case: the overlap was in fact almost complete. To increase the quality of the matching and reduce the impurity, we opted for nearest neighbour matching with replacement. This method enables more similar matched persons to be found (based on the propensity score), thus improving the quality of the matching. Unlike matching without replacement, this method also has the advantage that the results do not depend on the order in which the observations are matched.

The 240 PB-holders are the ‘experimental group’ and were matched with 206 respondents, forming the ‘control group’. One hundred seventy-nine PB-holders were matched with a unique respondent; 38 were matched twice, 18 three times and five were matched five times.

We performed three sensitivity analyses. As a first sensitivity analysis, we also carried out matching using an alternative approach to nearest neighbour matching, Mahalanobis matching. The second sensitivity analysis consisted of including community level covariates in the multilevel analysis, in line with Stuart [35]. The third sensitivity analysis excluded the variable ‘mastery’. The reason is that it may be argued that mastery is conceptually very close to independence.

## Results

Table 1 shows that, before the correction with propensity score matching, many background characteristics differed significantly between the two groups. In other words, people who opted for a PB had different background characteristics from people who opted for help in kind. After correcting with propensity score matching, no significant differences in background characteristics remained between the PB and help in kind groups. This, plus an average absolute bias of 4.7%, means that after propensity score matching the variables in the PB and help in kind groups are well balanced. The maximum average bias should be below 5% [26]. Hence, we were successful in matching the group of PB-holders to a group of non-PB-holders who were as similar as possible.

**Table 1:** Characteristics of respondents (personal budget and help in kind), uncorrected and corrected (*n* = 446)

Variable	averages				% bias	t-tests			
	uncorrected		after propensity score			uncorrected		after propensity score	
	personal budget	in kind	personal budget	in kind		*t*	*p*	*t*	*p*
Severity of physical disability	34.7	30.05	34.75	34.89	−1.8	−9.15	< 0.005	−0.2	0.84
Education	3.97	3.76	3.98	4.1	−7.3	−1.83	0.07	−0.76	0.45
Women (vs. men)	0.69	0.72	0.69	0.68	0.9	1.08	0.14	0.1	0.92
Living alone (vs. cohabiting)	0.55	0.56	0.55	0.53	4.2	0.28	0.39	0.46	0.65
Age	64.44	70.74	64.44	64.7	−1.7	6.31	< 0.005	−0.17	0.86
Income < 1000 euro (ref)									
Income 1000–1500 euro	0.22	0.15	0.23	0.16	16.1	−2.75	0.006	1.73	0.08
Income 1500–2000 euro	0.15	0.2	0.15	0.13	6.6	1.83	0.068	0.79	0.43
Income > 2000 euro	0.15	0.13	0.15	0.15	1.2	−0.8	0.42	0.13	0.90
No income data	0.14	0.12	0.13	0.14	−1.2	−0.75	0.45	−0.13	0.89
Mental health problem	0.23	0.16	0.23	0.26	−7.3	−2.59	0.009	−0.74	0.46
Mastery	13.93	15.53	13.9	13.82	2	5.68	< 0.005	0.21	0.83
% living alone 65+ in community	6.7	6.61	6.7	6.81	−9.8	−1.07	0.29	−1.05	0.29
% disabled in community	4.4	4.32	4.4	4.39	1.5	−1.29	0.20	0.17	0.86
% people below income threshold	6.34	6.3	6.36	6.47	−4.8	−0.27	0.79	−0.52	0.60
average absolute bias percentage					4.7				

The results of the multilevel analysis with propensity score matching do correspond with our hypothesis as far as the direction of the differences is concerned, but they are not statistically significant. People with a PB are thus no more or less independent, do not participate more or less and do not have a higher or lower quality of life than people receiving help in kind (Table 2). Without propensity score matching, the result would have been different: people with a PB would then have been found to participate less and experience a lower quality of life. These uncorrected results are, however, distorted by the fact that different people chose a PB than those who opted for help in kind (see Table 1 for the differences in averages, in the column ‘uncorrected’).

**Table 2:** Estimated values for the dependent variables (on a scale from 1 to 10) independence, participation and quality of life for respondents who have chosen a PB or help in kind (*n* = 446)

	sample	personal budget	in kind	difference	standard error	*t*	*p*
on independence	uncorrected	7.879	8.007	−0.128	0.103	−1.24	n.s.
	ATT multilevel	7.911	7.744	0.167	0.137	1.22	n.s.
on participation	uncorrected	6.629	6.940	−0.311	0.137	−2.28	sig.
	ATT multilevel	6.648	6.551	0.096	0.198	0.49	n.s.
on quality of life	uncorrected	7.538	7.889	−0.352	0.117	−3.01	sig.
	ATT multilevel	7.557	7.464	0.093	0.171	0.54	n.s.

Uncorrected: uncorrected for propensity score matching

ATT multilevel: corrected for propensity score matching (average treatment effect on the treated) and multilevel

As a sensitivity analysis, we also carried out Mahalanobis matching. The results were the same as for the nearest neighbour matching, i.e. no significant differences. A multilevel analysis with propensity score matching with the inclusion of community level covariates also did not produce different results. The third sensitivity analysis excluded mastery from the set of variables used in the calculation of the propensity score. This analysis is reported in Table 3. It turns out that in this sensitivity analysis people with a PB report significantly more independence than those receiving help in kind.

**Table 3:** Sensitivity analysis, excluding the variable mastery from the propensity score calculation: Estimated values for the dependent variables (on a scale from 1 to 10) independence, participation and quality of life for respondents who have chosen a PB or help in kind (*n* = 446)

	sample	personal budget	in kind	difference	standard error	*t*	*p*
on independence	uncorrected	7.879	8.007	−0.128	0.103	−1.24	n.s.
	ATT multilevel	7.947	7.610	0.149	0.137	2.26	0.024
on participation	uncorrected	6.629	6.940	−0.311	0.137	−2.28	sig.
	ATT multilevel	6.670	6.633	0.036	0.185	0.20	n.s.
on quality of life	uncorrected	7.538	7.889	−0.352	0.117	−3.01	sig.
	ATT multilevel	7.583	7.388	0.194	0.162	1.20	n.s.

Uncorrected: uncorrected for propensity score matching

ATT multilevel: corrected for propensity score matching (excluding mastery, average treatment effect on the treated) and multilevel

## Discussion

Our aim was to investigate whether receiving a PB coincides with more independence and social participation and a higher quality of life than receiving help in kind. This is not evident from our primary results. However, a sensitivity analysis, excluding the variable sense of mastery in the calculation of the propensity score, shows a significant result in the case of the dependent variable independence. This shows that the result for independence to some extent depends on the model specification and may be not so robust as to reject the hypothesis that having a PB coincides with more independence. However, based on this study, we conclude that on the whole it is not possible to demonstrate that, in terms of the outcome measures in our study, people receiving help purchased with a PB benefit more than people who receive help in kind.

What might explain the fact that we do not find a robust difference? Previous Dutch studies did not use propensity matching. Our uncorrected distribution of the independent variables matches the findings of previous studies; however, the outcome measures in the uncorrected data showed rather lower levels of independence, participation and quality of life, in contrast to the studies cited [10, 23]. This outcome is in line with more severe disability and people with mental health problems among PB users in our sample. As for the explanation of the lack of effects of PBs, PBs may not have the assumed beneficial effects on the studied outcomes. The effects of PBs may take time to develop [15]. Although there was only a relatively short period between the application for support and the interview (4–6 months), more than two thirds of the applicants already had a PB and applied for an additional type of support or a change in their support. We do not know how long they already had a PB. It may also be that, for some users, help in kind is just as ‘tailor-made’ as it would be with a PB. In countries where healthcare is less well organised than in the Netherlands, it is possible that PBs deliver added value on a larger scale. Another possible explanation is that PBs not always deliver customisation. People sometimes purchase the same home care package with a PB as they would receive with care in kind, resulting in little or no difference between a PB and help in kind. It is also possible that those who provide help to PB-holders are unable to do so at times that suit the recipient, or do not provide help in the way that the recipient would like. People with a PB may also hire people they know (e.g., relatives and friends) and find it difficult to state precisely what help they would like to receive and how. Also the quality of the care provided by people that are hired by the recipients of a PB and that provided by the suppliers of in-kind care, may not differ, as suggested in the literature [13].

It may be that customisation is not always an end in itself; it is known that people who apply for help from their local authority are sometimes encouraged to choose a PB because it is cheaper than providing help in kind. The local authority can apply lower rates for care purchased with a PB because there are fewer overheads than with home care organisations. However, the allocation of hours is independent of whether people choose for a PB or for care in kind. It is possible that some people choose a PB because they are encouraged to do so by a council official. In these situations, the choice may not have been based so much on a desire for more customisation of care, and the differences compared with recipients of care in kind are therefore small.

In this study we combined many different types of help. It is plausible that certain types of help are more important for someone’s independence, participation and/or quality of life than others. More intimate forms of help, for example personal care or nursing care, may have a bigger impact on quality of life than help with the household.

Information on the number of hours of help received was not available for this study. The more dependent people are on help and the more help they need, the more important it will be for them to be able to choose who helps them, with what and at what times.

The results of the multilevel analysis with propensity score matching are in the hypothesized direction, though they are not statistically significant in our primary analysis. This may be connected to the numbers in our analysis. If there was actually a difference in the population, we would need a minimum of 700 respondents to demonstrate this. However, given its size, it remains questionable whether this difference would have policy relevance. The sample size in our study was too small for sub-group analysis. Some groups, such as people with a physical handicap, may benefit more from PBs than others [5, 15, 16].

Our study was a secondary analysis of survey data that were collected for another purpose than assessing the effects of having a PB. As a consequence we had to do with limitations in the measurement of the outcome variables self-reported independence, participation and quality of life. A specific study to assess the effects of having a PB would have used more extensive and standardised measures for these concepts. In our view, the measurements of the outcomes is not optimal; however, using existing data to answer the question about the effects of PBs is better than not testing the basic assumptions about having a PB at all. Another limitation of the data used may be that the data were already collected in 2012. That would be a problem if the aim of the study was to describe the current situation in the Netherlands; however, we are testing an assumption about the effects of having a PB. The effects of having a PB may be context dependent and the context may have changed by now. Still, the assumption was refuted for the 2012 context in the Netherlands and this adds to our knowledge about the effects of PBs. The strength of our analysis is its methodological approach to make users and non-users of a PB as comparable as possible in an observational study.

Our finding that a PB is not robust for independence, and no more beneficial for participation and quality of life than help in kind, and findings from research in England, urges a research agenda that focuses at groups where bigger differences between PB and help in kind can be expected. For specific groups and in specific situations PBs can have great added value, for example for people with an unpredictable clinical picture, who sometimes need lots of help and sometimes little; for these people, a PB could be a more flexible solution than help provided via a bureaucratic organisation. People also sometimes have a disorder for which help is needed quickly and/or for which the recipient can only bear to be helped by someone they know. This may apply more often for people with a psychological, psychogeriatric or intellectual disability. Our sample contained only a small proportion of respondents with these disabilities. Subgroup analysis requires much larger numbers than we had in our sample. If subgroup analysis showed that PB-holders are indeed better off, then it would be a logical step to make PBs available in a much more targeted way. A second element of a research agenda would be more extensive research in international comparison of PB arrangements and the institutions in which they are embedded, combined with survey data that include effects of having a PB. PBs might work in some contexts and not in others. The supply and organisation of support differs between countries and so will the options for people that choose a PB, in terms of the range of service providers they can choose from, the flexibility of the service providers and the quality of their services. Insight in the transferability of effects of PBs from one context to another can help policy-makers as well as researchers.

## Conclusion

After propensity matching, no significant effects of having a personal budget were found on client-reported participation and quality of life, and a significant effect for independence was only found in a sensitivity analysis. A RCT would be stronger research design but not feasible in the Dutch situation. There may be several reasons for the lack of effect. First, perhaps there are no effects of PBs on client-reported independence, participation and quality of life. It is also possible that effects can only be found in specific situations and/or specific groups of clients. Therefore, more research into this is recommended.

## Data Availability

The data that support the findings of this study are available from The Netherlands Institute for Social Research – SCP but restrictions apply to the availability of these data, which were used under license for the current study, and so are not publicly available. Data are however available from the authors upon reasonable request and with permission of The Netherlands Institute for Social Research – SCP.

## Abbreviations

PB: Personal budget
RCT: Randomised controlled trial
